# fMRI Evidence for the Involvement of the Procedural Memory System in Morphological Processing of a Second Language

**DOI:** 10.1371/journal.pone.0097298

**Published:** 2014-05-12

**Authors:** Christos Pliatsikas, Tom Johnstone, Theodoros Marinis

**Affiliations:** 1 Centre for Integrative Neuroscience and Neurodynamics, University of Reading, Reading, United Kingdom; 2 Department of Clinical Language Sciences, School of Psychology & Clinical Language Sciences, University of Reading, Reading, United Kingdom; 3 Department of Psychology, School of Psychology & Clinical Language Sciences, University of Reading, Reading, United Kingdom; IIT - Italian Institute of Technology, Italy

## Abstract

Behavioural evidence suggests that English regular past tense forms are automatically decomposed into their stem and affix (played = play+ed) based on an implicit linguistic rule, which does not apply to the idiosyncratically formed irregular forms (kept). Additionally, regular, but not irregular inflections, are thought to be processed through the procedural memory system (left inferior frontal gyrus, basal ganglia, cerebellum). It has been suggested that this distinction does not to apply to second language (L2) learners of English; however, this has not been tested at the brain level. This fMRI study used a masked-priming task with regular and irregular prime-target pairs (played-play/kept-keep) to investigate morphological processing in native and highly proficient late L2 English speakers. No between-groups differences were revealed. Compared to irregular pairs, regular pairs activated the pars opercularis, bilateral caudate nucleus and the right cerebellum, which are part of the procedural memory network and have been connected with the processing of morphologically complex forms. Our study is the first to provide evidence for native-like involvement of the procedural memory system in processing of regular past tense by late L2 learners of English.

## Introduction

According to dual systems of morphological processing, English past tense verbs are processed according to their regularity by native speakers [1]. Based on a linguistic rule, regular inflections (e.g. *played*) undergo an obligatory outstripping of the -*ed* suffix during online processing, in order for the stem *play* to be accessed. As such, this rule does not apply to idiosyncratically formed irregular inflections, such as *kept*. Ullman [2] built on this distinction to suggest differentiated brain networks for the two types of inflection: regular inflection is an automated procedure that is subserved by what he called the “procedural memory system”, which includes the left Inferior Frontal Gyrus (LIFG), the basal ganglia and the cerebellum. This system is irrelevant for the processing of the idiosyncratic irregular forms, which are subserved by the “declarative system”, involving parahippocampal areas. In the same model, Ullman suggested that this distinction does not apply to late L2 learners of English. This is because of maturational constraints in the procedural system, which prevent the learners from establishing the implicit rule. Consequently, according to Ullman, L2 learners should process regular forms similarly to irregular ones, i.e. by utilising the declarative system. This section reviews the available evidence for morphological processing in the brain of native and non-native speakers of a language.

Processing of inflection by native speakers has been studied with fMRI using priming tasks. Marslen-Wilson & Tyler [3] reviewed a number of neuroimaging studies and defined a frontotemporal network that becomes activated for the processing of regular inflection, which includes the LIFG. Most of these studies employed auditory tasks [4]–[6]. However, there is limited evidence on the visual processing of morphologically complex forms, and most fMRI studies to date investigated processing of derivational morphology. Devlin, Jamison, Matthews and Gonnerman [7] tested participants in a masked priming task which included morphological word pairs using derivational morphology (*hunter-hunt*) that were compared to semantic (*sofa-couch*), orthographic (*passive-pass*) or unrelated (control) pairs (*award-much*). Devlin et al. observed a reduction in the activity of the left posterior occipitotemporal cortex for both morphological and orthographic pairs compared to control, and a signal reduction in the left middle temporal area for both semantic and morphological pairs, compared to control, but no effects that were specific to morphological pairs. Based on this observation, Devlin et al. suggested that morphology is not a fundamental linguistic component, but it emerges as a convergence of meaning and form. Gold and Rastle [8] also investigated derivational morphology using a masked priming task on the processing of pseudomorphological pairs, i.e. pairs that were semantically or morphologically unrelated, but the monomorphemic prime appeared to contain two valid morphemes, namely the target and a derivational suffix such as -*er* (*archer-arch*).They compared them to orthographic (*pulpit-pulp*), semantic (*forest-tree*) and unrelated pairs (*stamp-iron*). Gold and Rastle did not find any effects in the LIFG for their pseudomorphological pairs, and suggested that the LIFG is the site of later strategic components of morphological analysis, which cannot be unveiled by masked priming. However, the lack of effects in the LIFG for the pseudomorphological pairs may simply indicate that the LIFG is involved only in the processing of real inflections or derivations.

In contrast to the two previous studies, Bozic, Marslen-Wilson, Stamatakis, Davis and Tyler [9] highlighted the role of the LIFG in the processing of derivational morphology. This study used a delayed repetition priming task; the experimental pairs were either real derivations (*bravely-brave*) or pseudoderivations (*archer-arch*), compared to identical (*mist-mist*), semantic (*accuse-blame*) and orthographic pairs (*scandal-scan*). Bozic et al. observed increased activity in the LIFG for the first presentation of complex (derivations and pseudoderivations) forms, compared to simple forms; the LIFG activity was significantly reduced for the second presentation of morphologically related words, compared to unrelated words, demonstrating a delayed priming effect. According to Bozic and colleagues, this finding suggested the preferential engagement of the LIFG in the processing of morphologically complex words, even in cases of pseudoderivations with recognisable morphemes. However, it is unclear why similar effects were not observed by Devlin et al. [7].

Apart from the involvement of the LIFG, some evidence has been provided for the role of the basal ganglia in morphological processing. Vannest, Polk and Lewis [10] used an encoding task, in which lists of words were presented in a blocked fMRI design, followed by recognition tests. Vannest and colleagues presented English inflections and derivations, along with non-decomposable forms, and reported activations for complex vs. simple words, not only in the LIFG but also in the bilateral caudate nucleus. This basal ganglia involvement in morphological processing has also been reported in research with Italian speakers [11].

To conclude, the available fMRI studies on visual morphological processing describe a network of brain regions that is involved in the processing of complex derivations in English; however, only Vannest et al. investigated processing of inflection. The application of inflectional rules may involve different neural substrates compared to the processing of derivation [12]. The effects of rule application have been demonstrated by auditory priming fMRI studies; however, while in auditory studies the various constituents of a complex word become available in a serial manner, in visual tasks all components become available simultaneously. Therefore, it is important to investigate how rule application takes place in a visual task, and whether the findings are in line with existing results on the processing of derivational morphology.

Despite the existing theoretical models on the processing of inflection by L2 learners [2], and despite recent behavioural evidence in favour of dual-route processing in L2 [13], there is a dearth of research on the neurological correlates of inflectional processing in L2 English. Some evidence is available on how speakers of other languages process inflection in their L2. Lehtonen and colleagues [14] tested early Swedish-Finnish bilinguals in a lexical decision task, which included complex inflections and simple monomorphemic nouns in Swedish and in Finnish, yielding four conditions. They reported increased activation in the LIFG for complex Finnish inflections only, compared to the other three types, which additionally did not differ from each other. Lehtonen and colleagues interpreted this finding as evidence for a dual-route system for the highly-inflected Finnish, where complex forms are processed via rule-application and simple forms are directly retrieved, and a single-route system of direct retrieval for Swedish; notably, bilinguals appear to utilize both systems. However, since these participants were early L2 learners, this finding is not sufficient to suggest that all L2 learners will show native-like patterns of brain activity when it comes to L2 inflection. Therefore, it is crucial to study also the processing routines of late L2 learners.

To the best of our knowledge, no fMRI studies to date have investigated dual-route processing by late L2 learners. However, some evidence comes from the ERP literature: Hahne, Mueller and Clahsen [15] tested Russian learners of L2 German, in a task presenting German participles embedded into sentences. German features regular (*tanzen* (dance) - *getanzt* (danced)) and irregular participles (*laufen* (walk) –*gelaufen* (walked)). In their experiment, Hahne et al. attached the regular morpheme to irregular verbs, and vice versa, in order to create “irregularised” (e.g. *getanzen) and “regularised” (e.g. *gelauft) non-word participles, respectively. Regularisations elicited an ERP pattern known to underlie misapplication of morphological rules by native speakers (a LAN effect followed by a P600) [16]; irregularisations elicited an ERP effect (an N400) that characterizes lexico-semantic processing [17]. Hahne et al. interpreted this finding as indicative of native-like dual-route processing suggesting that L2 learners utilised a grammatically-informed route for the processing of rule misapplications (regularisations), and a semantically-informed route for the processing of non-words that were not created based on the default rule (irregularisations). These findings confirm Ullman's [1], [2] prediction for a dual-route in morphological processing. Based on this, it is important to investigate whether the native-like processing that emerges from the ERP findings is also reflected in the same networks being activated in L2 learners using fMRI.

Some recent evidence for the role of the procedural system in L2 morphological processing was presented by Pliatsikas, Johnstone and Marinis [18]. Pliatsikas et al. performed a Voxel-Based Morphometry (VBM) analysis on structural data of Greek L2 learners and native speakers of English, and reported greater Grey Matter (GM) volume in the L2 learners in a right cerebellar region already shown to be involved in grammatical processing [19]. Importantly, the GM volume in L2 learners correlated positively to their speed in processing regular inflections e.g. *played*) in a masked priming task, suggesting that the greater the GM volume the faster they processed the inflected forms. No similar correlations were reported for the processing of irregular forms (e.g. *kept*) by L2 learners, or for the processing of either type of inflection by native speakers. This suggests a dynamic restructuring of the cerebellum in L2 learners in order to acquire and/or accommodate the L2 morphological rule.

This fMRI study aims to investigate whether the proposed distinction between processing regular and irregular verbs [1] has its equivalents in brain activity of native and late non-native speakers of English. To do that, we examined brain activity in the subjects from [18] that were tested in the masked priming task with regular and irregular verbs. Critically, we focused on the brain areas suggested by Ullman to underlie processing of regular inflection, namely the LIFG, basal ganglia and the cerebellum.

As far as natives speakers are concerned, we predicted that the differential processing of regular vs. irregular verbs would engage the LIFG-basal ganglia-cerebellum network, which has been described as the site of procedural memory [2], [4], [20]. A similar prediction was drawn for the late L2 learners, based on the results from [15]: if the dual-route system is available to the late L2 learners, they are expected to engage a similar brain network for the processing of regular vs. irregular verbs.

## Methods

### 1.1. Ethics statement

This research was approved by the University of Reading Research Ethics Committee. All participants provided written informed consent prior to participating.

### 1.2. Participants

The participant groups from Pliatsikas et al. [18] were also tested in this experiment: 17 Greek-English L2 learners (12 female) with naturalistic exposure to an English-speaking environment (L2 group, *M*age: 27.5, range: 19–37, *SD*: 5.55) and 22 English native speakers (15 female) (NS group, *M*age: 24.5, range: 20–38, *SD*: 3.9). The two experiments were run as part of the same testing session. The L2 learners started learning English after the age of 6 (*M*age of onset: 7.7, range: 6–14, *SD*: 2.2), and were therefore classified as late L2 learners of English. The participants were recruited from the University of Reading and were awarded with a monetary reward. All L2 participants reported English as the foreign language they spoke the best, and they were assessed for their proficiency in English with the Quick Placement Test [21]. Their average score was 82.4% (range: 70–100%, *SD*: 10%, Effective-Mastery proficiency level). They also reported the years they had lived in the UK (*M*naturalistic exposure = 3.97 years, *SD* = 3.53), the amount of years they spent learning English (*M* = 9.29, *SD* = 3.46), and the percentage of daily use of English (*M* = 51.7, *SD* = 21.6).

### 1.3. Materials & Design

**Figure 1:**
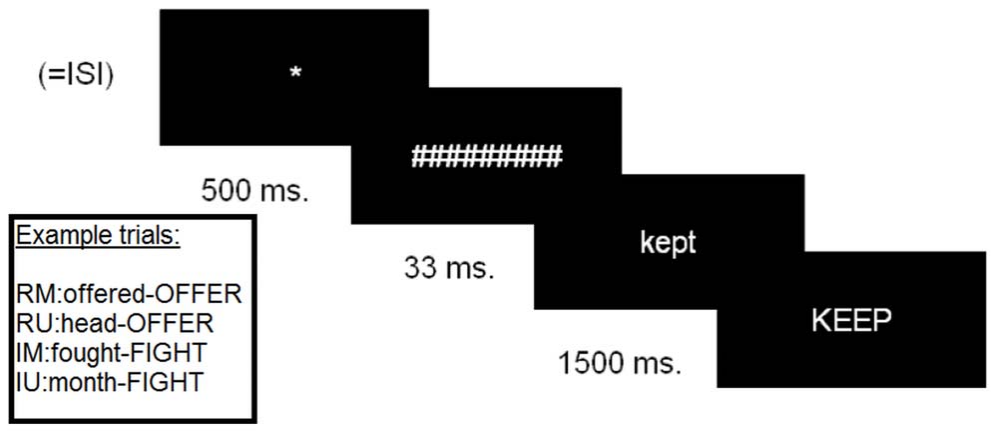
Schematic representation of the masked priming task with example pairs per condition. ISI: Interstimulus Intervals.

The materials consisted of regular and irregular verbs as targets, paired with either their past tense forms or an unrelated word as a prime, therefore creating four experimental conditions of 20 items each: Regular Morphology (RM) (*played-play*), Regular Unrelated (RU) (*fork-play*), Irregular Morphology (IM) (*kept-keep*) and Irregular Unrelated (IU) (*fork-keep*). In each trial a mask (#####) was presented for 500 ms., followed by the prime in lower case for 33 ms., followed by the target in upper case for 1500 ms. The task also included an equal number of nonword targets, paired with nonword primes, and the task was a lexical decision on the target. Of the four experimental conditions, only RM is considered to involve rule application, and therefore engage the LIFG-basal ganglia network. For more details, see Pliatsikas et al. [18]. For the purposes of the fMRI investigation we created an event-related fMRI design with variable Interstimulus Intervals (ISI). The experimental trials were pseudorandomised, and a fourth visual event was added before each trial in the form of a star (*), with duration equal to the ISI preceding each trial. The masked priming task is illustrated in Figure 1.

### 1.4. Procedure

The experiment was designed and presented through the E-prime experimental software [22], [23], which also collected reaction time (RT) and accuracy data per trial. Stimuli were presented with a NordicNeurolab Visual System (SVGA, resolution: 800 (x3)×600, 16.7 million colours, refresh rate: 75 Hz, field of view (FOV): 30^o^ horizontal, 23^o^ vertical). All stimuli were presented in white characters (font: Courier New, size: 18pts) against black background.

Prior to the scan, the experimental task was explained to the participants. They were given an MRI-compatible 4-button fORP response pad (Current Designs, Inc.) with two active buttons, one for the YES responses and one for the NO responses of the lexical decision task. The instructions were also projected in written form immediately prior to the scan, followed by a practice run. At the end of the practice run, the participants had the opportunity to ask questions about the experiment. The experiment lasted 10 minutes.

### 1.5. fMRI data acquisition

Whole-brain functional and anatomical images were acquired using a 3.0 Tesla Siemens MAGNETOM Trio MRI scanner with Syngo software and 12-channel Head Matrix coil. Functional images were acquired using a T2*-weighted gradient-echo echo planar imaging (EPI) pulse sequence with 30×4 mm axial slices, interleaved from bottom to top (interslice gap: 1 mm, TE: 30 ms., TR: 2000 ms., flip angle: 90^o^, FOV: 192×192 mm, in-plane matrix resolution: 64×64). High-resolution T1-weighted MP RAGE gradient-echo anatomical images were collected with 176×1 mm slices (TE: 2.52 ms., TR: 2020 ms., TI:1100 ms., FOV.: 250×250 mm, image matrix resolution: 256×256). Finally, a field map was acquired with 30×4 mm slices (TE: 4.92/7.38 ms., TR: 488 ms., FOV: 192×192 mm). The data are available upon request.

### 1.6. fMRI data analysis

All data processing was carried out using FEAT Version 5.98, part of FSL [24], [25]. Non-brain tissue was removed from the images using BET [26]. The functional data were motion-corrected using MCFLIRT [27], and slice-time corrected using Fourier-space time-series phase-shifting. To correct for image distortion, fieldmap-based EPI unwarping was applied by using PRELUDE+FUGUE [28], [29] (Effective echo spacing: 0.7 ms., EPI TE: 30 ms., unwrap direction: y, 10% signal loss threshold). In addition, the images were spatially smoothed using a Gaussian kernel with a Full Width at Half Maximum (FWHM) value of 8 mm, and grand-mean intensity normalisation of the entire 4D dataset by a single multiplicative factor was applied. Finally, highpass temporal filtering was applied (Gaussian-weighted least-squares straight line fitting, with sigma = 45.0 s).

Individual participant data were analysed using a general linear model, with the responses to the four experimental conditions modelled as separate Explanatory Variables (EVs). Another four EVs modelled the nonword conditions as events of no interest, and a final EV modelled the errors and the missed responses, and was orthogonalised to the four experimental EVs. A boxcar waveform that modelled the actual onset and duration of each stimulus, as provided by the RT data was convolved with a double-Gamma hemodynamic response function (HRF) to create each EV. The same temporal filtering was applied to the model that was applied to the data, and the model was completed with the addition as separate regressors of EV temporal derivatives, in order for the model to better fit the time course of the actual data acquisition, and motion regressors, as estimated by MCFLIRT during preprocessing [30]. Time-series statistical analysis was carried out using FILM with local autocorrelation correction [31], [32].

To examine the main effects of regular and irregular inflectional processing, the contrast between the Morphology and the Unrelated conditions for each type of verb was calculated. This gave us the following contrasts: Regular Morphology>Regular Unrelated (RM>RU) and Irregular Morphology> Irregular Unrelated (IM>IU). Additionally, in order to investigate the differences between Regular and Irregular inflectional processing, the contrast between the Morphology conditions was calculated, giving us the following contrasts: Regular Morphology>Irregular Morphology (RM>IM) and Irregular Morphology>Regular Morphology (IM>RM). The estimated contrasts, along with the EVs themselves, gave a total number of 8 contrast images for each participant. The contrast images were registered to the 152-brain T1-weighted Montreal Neurological Institute (MNI) template using FLIRT [27], [33] in a two-stage process: first, for each participant an example fMRI low resolution image was registered to the same participant's high resolution T1-weighted structural image by using a 7 DOF (degrees of freedom) linear transformation. Second, the high resolution image was registered to the standard MNI template by using a 12 DOF linear transformation. These two transformations were subsequently combined into a third one, which was used for the registration of the low resolution fMRI images into the standard space prior to group analyses.

In the between-groups analysis the same contrasts were analysed using a mixed effects model in FLAME, stages 1 and 2 [34]–[36]. We restricted our analysis to those areas that have been previously linked to morphological processing, namely the LIFG, including BA44 and BA45, and the basal ganglia, including bilateral amygdala, globus pallidus, putamen and caudate nucleus. We created masks of the regions of interest based on the Juelich Anatomical Atlas [37]. We also investigated activation of the cerebellar cluster reported in Pliatsikas et al. [18] by creating another mask. Each of the three images was applied as a pre-thresholding mask in separate between-group analyses in FSL, resulting in three separate analyses in total. The resulting statistic images from the higher level analyses were thresholded using images determined by Z>2.3 and a corrected cluster significant threshold of p = 0.05. A whole-brain analysis can also be found in Text S1.

## Results

Two participants from the NS group and one from the L2 group were excluded from the fMRI analysis due to excessive head movement, defined as any displacement above 3 mm from the position of the reference image. The following section illustrates the behavioural results of the experiment, followed by the fMRI findings for each of the areas of interest.

### 1.1. Demographics

The two groups did not differ significantly in terms of age [F(1,34) = 3.423, p = 0.073]. Additionally, a Fischer's Exact test revealed that there was no significant differences in gender distribution between the two groups (p = 0.647).

### 1.2. Accuracy

Table 1 illustrates accuracy figures per group and per condition. A mixed three-way ANOVA with two within-groups factors, Verb Type (regular, irregular) and Condition (morphology, unrelated), and Group (NS, L2) as the between-groups factor did not reveal a significant main effect of Group [F(1,34) = 3.249, p = 0.08, η^2^ = 0.087], Verb Type [F(1,34) = 3.434, p = 0.073, η^2^ = 0.092] or Condition [F(1,34) = 0.849, p = 0.363, η^2^ = 0.024]. Additionally, none of the interactions was significant: Verb Type x Condition [F(1,34) = 1.475, p = 0.233, η^2^ = 0.042], Group x Verb Type [F(1,34) = 0.084, p = 0.773, η^2^ = 0.002], Group x Condition [F(1,34) = 2.813, p = 0.103, η^2^ = 0.076], and Group x Verb Type x Condition [F(1,34) = 0.333, p = 0.567, η^2^ = 0.010]. Only the correctly answered trials were retained for the subsequent fMRI analyses.

**Table 1:** Accuracy % (SD) per group and per condition.

	NS	L2
Regular Morphology	96.3 (3.9)	95.6 (6.5)
Regular Unrelated	96.3 (5)	92.5 (8.4)
Irregular Morphology	97.3 (3.8)	95.3 (6.9)
Irregular Unrelated	98.2 (2.9)	95.0 (4.8)

### 1.3. Reaction times

Only RTs for real word targets were analysed. RTs were screened for extreme values defined as any RT below 100 ms. No upper limit was defined because the responses were limited by the design to a maximum of 1500 ms. No extreme values were found. Additionally, the data were screened for outliers, defined as values that lay beyond 2 standard deviations from the mean RT for each condition per subject and per item. 6.03% of the NS data and 8.7% of the L2 data were identified as outliers and were subsequently replaced by the subject or item mean RT per condition. Table 2 illustrates the group means per experimental condition.

**Table 2:** Mean RTs (SD) per group and per condition.

	NS			L2		
	Morphology	Unrelated	U-M	Morphology	Unrelated	U-M
Regular	538 (34)	543 (42)	5	580 (49)	568 (39)	−12
Irregular	530 (41)	553 (37)	23**	558 (42)	601 (49)	43**
I-R	−8	10		−22*	33**	
* p<0.05** p<0.001						

A Shapiro-Wilk test revealed that our RT data per group and per condition were normally distributed (all ps>0.3), and therefore we proceeded with parametric tests. In order to investigate for differences in the mean RTs per condition between the two groups, a mixed three-way ANOVA was conducted with two within-subjects factors, Verb Type (Regular, Irregular) and Condition (Morphology, Unrelated), and one between-subjects factor, Group (NS, L2). The analysis revealed a main effect of Condition [F(1,34) = 21.06, p<0.001, η^2^ = 0.382], a main effect of Group [F(1,34) = 7.996, p = 0.008, η^2^ = 0.190], a significant Condition x Verb Type interaction [F(1,34) = 33.039, p<0.001, η^2^ = 0.493], and a significant Group x Condition x Verb Type interaction [F(1,34) = 8.001, p = 0.008, η^2^ = 0.160]. In order to unpack the three-way interaction, we analysed the data separately for each group with a repeated-measures two-way ANOVA, with the factors Condition and Verb Type.

For the L2 group the analysis revealed a main effect of Condition [F(1,15) = 6.685, p = 0.021, η^2^ = 0.308] and a significant Condition x Verb Type interaction [F(1,15) = 29.753, p<0.001, η^2^ = 0.665]. Subsequent paired samples T-Tests revealed that IM had significantly shorter RTs than IU [t(15) = −7.642, p<0.001)] and RM [t(15) = −2.170, p = 0.047)], and that RU had significantly shorter RTs than IU [t(15) = −4.565, p<0.001)].

For the NS group the same analysis revealed a significant main effect of Condition [F(1,19) = 19.208, p<0.001, η^2^ = 0.503] and a significant Condition x Verb Type interaction [F(1,19) = 5.262, p = 0.033, η^2^ = 0.217]. Paired samples T-Tests revealed that this interaction was due to IM having shorter RTs than IU [t(19) = −4.402, p<0.001)], suggesting a priming effect for irregular verbs. No other significant effects were found. The behavioural results are also illustrated in Figure S1.

### 1.4. fMRI findings

The between-groups analysis revealed no significant differences between the two groups in the areas under investigation and for all contrasts of interest. This suggested that there are no differences in the way that our native and highly proficient non-native speakers of English process inflection. Therefore, in order to investigate the brain activity elicited by the processing of inflection, we consider here the main effect results across all participants in all contrasts of interest.

Significant activation was seen for the RM>IM contrast in two regions within the LIFG, pars opercularis. Additionally, activation was observed in bilateral caudate nucleus and the right cerebellum. No significant activations were revealed for the IM>RM contrast. Furthermore, for the RM>RU contrast there was activation of the LIFG, pars opercularis, but not the basal ganglia or the cerebellum, and for the IM>IU contrast there was activation of the left cerebellum. The significant activations per contrast for the combined group appear in Table 3.

**Table 3:** Significant peak activations of the combined group for the contrasts of interest.

Contrast	hemi	region	Cluster size^a^	Z	x	y	z
RM>IM	L	IFG oper.^b^	273	3.4	−43.6	7.47	25
	L	IFG oper.	236	4.14	−46.1	9.92	−0.247
	R	caudate	273	3.95	15.4	10.9	7.51
	L	caudate	124	2.85	−12.5	6.98	8.7
	R	cerebellum	78	3.25	33.6	−76.1	−43.1
RM>RU	L	IFG oper.	523	3.3	−43.6	14.9	19.5
IM>IU	L	cerebellum	98	3.52	−20.3	−82	−19.9

All coordinates in MNI space.

a Cluster size is expressed in number of 3×3×4 mm voxels.

b IFG oper: Inferior Frontal Gyrus, pars opercularis.

**Figure 2:**
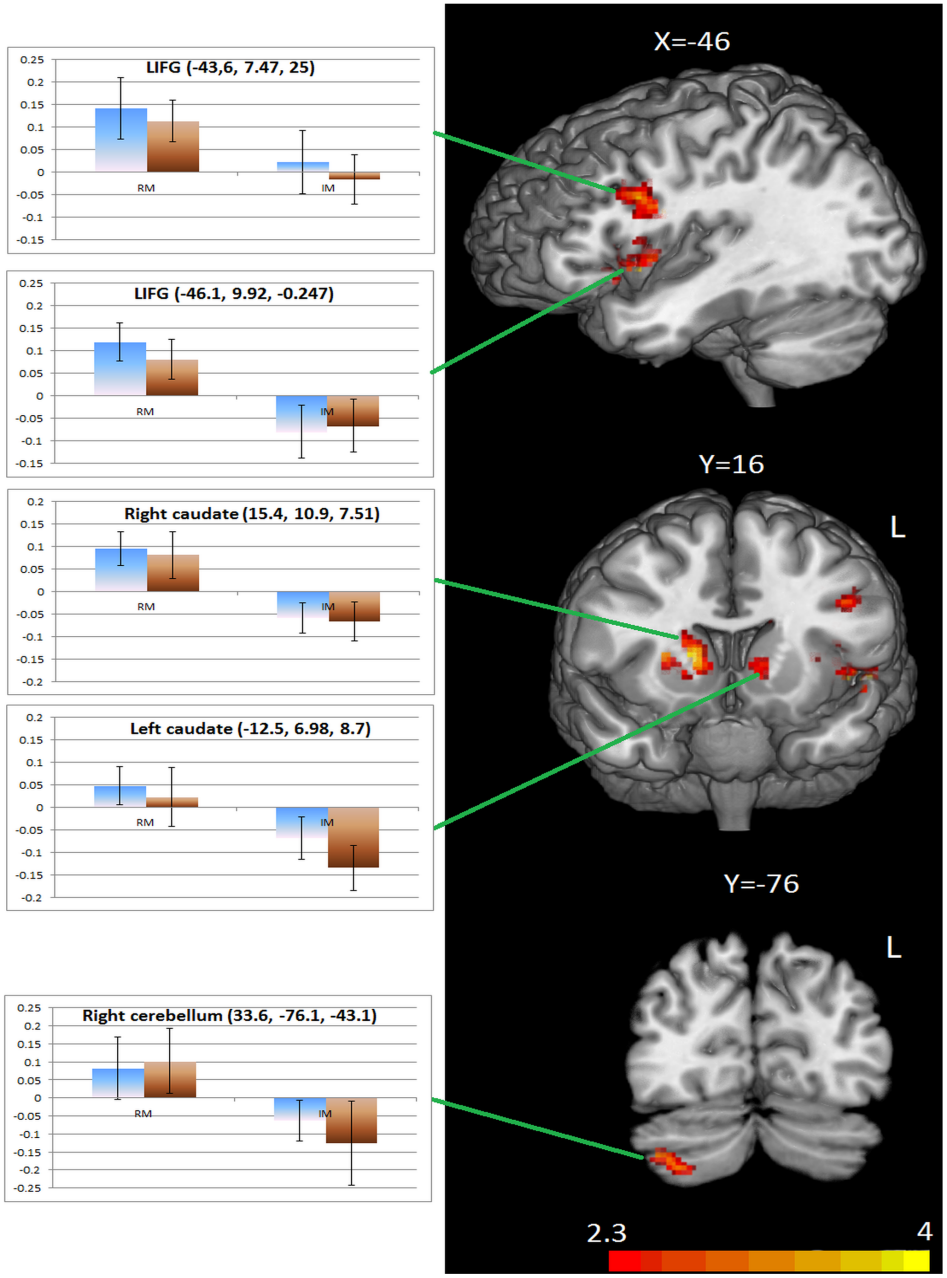
Significant activations for the Regular Morphology > Irregular morphology contrast across the combined participant group. From top to bottom: LIFG, bilateral basal ganglia, right cerebellum. The activations are expressed in GRF-corrected Z values. The bar charts illustrate percent signal change in brain activity per condition and per group (blue: L2, brown: NS) for each of the significantly activated clusters. The error bars represent the standard error of mean.

The group analysis findings confirm the involvement of the LIFG in the processing of morphologically complex forms, and also provide evidence for the role of bilateral caudate nucleus and the right cerebellum. Additionally, the findings indicate the absence of any between-groups differences. Activations for the RM>IM contrast are illustrated in Figure 2, overlaid on a standard brain template for illustrative purposes.

### 1.5. Effects of L2 linguistic background

In order to investigate whether the linguistic background of the L2 learners affects their processing of inflection, we ran a series of correlations between the background data of our participants (proficiency, exposure, AoA, daily use of English, years of learning English) and (a) the behavioural results and (b) the brain activity across the activated areas, calculated as the difference between RM and IM.

In terms of accuracy in the task, none of the above measures was significantly correlated with the participants' accuracy per condition, as well as overall accuracy (all ps >0.1). In terms of RTs, only Proficiency revealed significant negative correlation with RU [r(16) = −0.521, p = 0.038], IM [r(16) = −0.515, p = 0.041] and IU[r(16) = −0.531, p = 0.034], but not with RM [r(16) = −0.360, p = 0.171], although the trend is in the similar direction to the other conditions. This suggested that the more proficient readers were responding faster than the less proficient readers.

In terms of the BOLD activity, there was a significant correlation between naturalistic exposure and the RM-IM difference in the inferior LIFG cluster [r(16) = −0.584, p = 0.018], and between proficiency and the RM-IM difference in the left caudate [r(16) = −0.504, p = 0.046]. No other correlations were significant (all ps > 0.1). These findings suggest that L2 learners with increased L2 naturalistic exposure and/or proficiency demonstrate increased recruitment of the procedural network for the processing of regular inflections.

## Discussion

This study implemented a masked-priming task into an fMRI experiment in order to identify the neural correlates of morphological processing, and also to investigate any differences between native and highly proficient late non-native speakers in the processing of English past tense inflection. This section discusses the behavioural results, followed by the observed effects in each brain area of interest in relation to theories about morphological processing in L1 and L2.

Our behavioural results revealed priming effects for Irregular verbs only, for both of our groups. Additionally, RM produced longer RTs for the L2 group only. This is not a novel result: in a previous study [38] we also observed strong priming effects for irregular verbs for both NS and L2 groups, and also a significant inhibition for RM for the L2 group only. The replication of this pattern reinforces our previous suggestion that the application of morphological rules may be a costly procedure for non-native speakers; however, it also appears that increased proficiency may be beneficial for morphological processing in L2 learners, but this effect did not reach statistical significance. Nevertheless, the small sample of the present study does not permit us to draw any strong conclusions from the behavioural findings.

In line with previous findings [3], our groups did not differ in demonstrating increased activity in the LIFG for the processing of regular pairs compared to irregular pairs. Ullman [2] has described the LIFG as part of the procedural memory network which subserves the application of grammatical rules. In this light, the increased activity in the LIFG is likely to reflect the automatic application of the past tense rule for regular pairs such as *played-play*, which is not applicable to irregular pairs, such as *kept-keep*. Notably, in our findings this pattern was common across groups, suggesting that rule-based decomposition of inflections applies to native and highly proficient late non-native speakers alike, refuting Ullman's [2] suggestions of an under-developed procedural system in L2 learners.

Table 3 shows that the RM>IM contrast activated two distinct regions in the LIFG. The first, more inferior, activation peaked at -46.1, 9.92, −0.247. Increased activation in this area was also reported in [9] for the initial presentation of morphologically complex primes, involving derivation, and was interpreted as evidence for the LIFG involvement in the processing of morphologically complex words. Our findings also show increased activity in this region for regular verb pairs, compared to irregular verb pairs. This indicates that activation in this region signifies the decomposition of morphologically valid complex forms not only for derivation, but also for inflection. It is less likely that this effect signifies morphological priming, since priming effects are normally demonstrated with reductions to the brain activity for morphological pairs, compared to unrelated pairs (also in [9]). It is possible that the proximity of the two processes (decomposition and target recognition) in our experiment has obscured any priming effects in this region.

The second, more superior, activation in the LIFG peaked at −43.6, 7.47, 25. This region has previously been linked to the processing of regular morphological pairs, compared to irregular ones [6]. Therefore, and based on our findings too, this region appears to be specifically involved in the application of the past tense rule during processing, irrespectively of the modality. The same LIFG region was also activated for the RM>RU contrast, and this finding constitutes further evidence that the effects in this region are related to processing of the morphologically complex prime.

The explanation of the LIFG effects as indicative of the application of the past tense rule may also explain the lack of LIFG effects in studies that used a visual masked priming task with derivations [7], [8]. There are several differences between derivation and inflection: derivation creates new lexical entries, whereas inflection does not; derivation may or may not preserve the semantic relationship to their roots (compare *adjust-adjustment* to *depart-department*), whereas inflection always does; derivation often changes the syntactic category of words (e.g. verb to noun), whereas inflection does not. Based on these differences, it is not surprising that the effects that Devlin et al. [7] reported for derivational pairs overlap those for orthographic and semantic pairs: processing of *hunter* facilitates the recognition of *hunt* because of the activation of the semantic and orthographic properties of the two forms. In our experiment, although both types of verb pairs maintain a strong orthographic and semantic relationship, the morphological rule is applied only in the regular pairs and elicits increased activity in the LIFG, compared to irregular pairs.

Further in line with Ullman's model, the comparison of regular vs. irregular morphological pairs activated the caudate nucleus bilaterally. The right caudate nucleus has been previously linked to processing of derivation by healthy participants in Italian [11], while Vannest and colleagues [10] showed increases in activity in bilateral caudate nucleus for both inflected and derived forms, compared to non-decomposable forms. These findings, in conjunction with our results, suggest that the caudate nucleus is implicated in the automatic decomposition of valid complex forms, both inflections and derivations.

A further finding of this study is the activation of the right cerebellum for the processing of regular vs. irregular morphological pairs. Notably, the observed activation is located in an area that has been shown to be activated for language-related tasks [19], and has also more recently been suggested to underlie morphological learning and processing in L2 [18]. A few studies have proposed a role of the cerebellum for grammatical processing [39]–[43], and Ullman [2] has included it in the procedural network. Our results, in conjunction with the findings from Pliatsikas et al. [18] that were acquired from the same group of subjects, suggest that the cerebellum is an important structure for L2 grammatical learning and processing: not only the learning of a grammatical L2 rule may be related to structural changes in the cerebellum, but also the cerebellum, along with the rest of the procedural network, is active for the processing of regular inflection by native speakers and L2 learners. The activation of the left cerebellum for the IM>IU contrast is harder to interpret: IM pairs bear a semantic relationship which does not apply to IU pairs, and this may be the cause of the significant difference. However, this effect needs to be taken with caution, as the available evidence suggests that semantic language tasks engage the right cerebellum in right handed participants [19]; moreover, if this activation is related to the semantic relationship between the prime and the target, we should expect to see it for the RM>RU contrast too. Therefore, we cannot draw any strong conclusions from this effect.

An important finding of our investigation is the absence of any between-groups differences in the processing of regular vs. irregular inflection. This applied to all three areas of interest, which form part of the procedural network, as defined by Ullman [2]. Since the procedural network in this study was shown to be engaged in the processing of regular inflection, as predicted, we can deduce that late L2 learners have the same rule-application combinatorial skills as native speakers of English. This is not surprising: behavioural [13] and ERP [15] evidence have suggested native-like morphological processing in late adult second language learners. Our study confirms this by providing further evidence from fMRI data. This body of evidence challenges the maturational constraints proposed by the declarative/procedural model, and suggests that other factors, such as proficiency of linguistic immersion, may be crucial for the acquisition of L2 morphology. Our results provide some preliminary evidence for the effects of these factors: the difference in processing regular vs. irregular forms was greater in the LIFG as immersion increased, and also greater in the left caudate as proficiency increased. This suggests that exposure and/or proficiency may lead to increased usage of the procedural system for morphologically complex forms, and more generally, to more efficient L2 grammatical processing [13], [44]. Future neuroimaging studies should aim to manipulate these factors more carefully, for example by comparing low- to high-proficient L2 learners, or L2 learners with and without naturalistic exposure to the L2.

To conclude, the activation of the LIFG, the caudate nucleus and the cerebellum for the processing of morphologically complex forms confirms that the procedural network is involved in morphological processing [2]. Moreover, our study provides evidence for the first time that the same network is also involved in morphological processing by highly proficient late non-native speakers.

## Supporting Information

Figure S1 **Behavioural results per group and per condition.** Top: Accuracy (%), bottom: Reading times (msec). The error bars represent the standard error of the mean.
(TIF)

Text S1 **fMRI whole-brain analysis.**
(DOCX)
