# Supplementary figures and images for: fMRI Evidence for the Involvement of the Procedural Memory System in Morphological Processing of a Second Language
Source: PLoS One. 2014 May 12;9(5):e97298. doi: 10.1371/journal.pone.0097298 (PMC4018348; doi:10.1371/journal.pone.0097298)

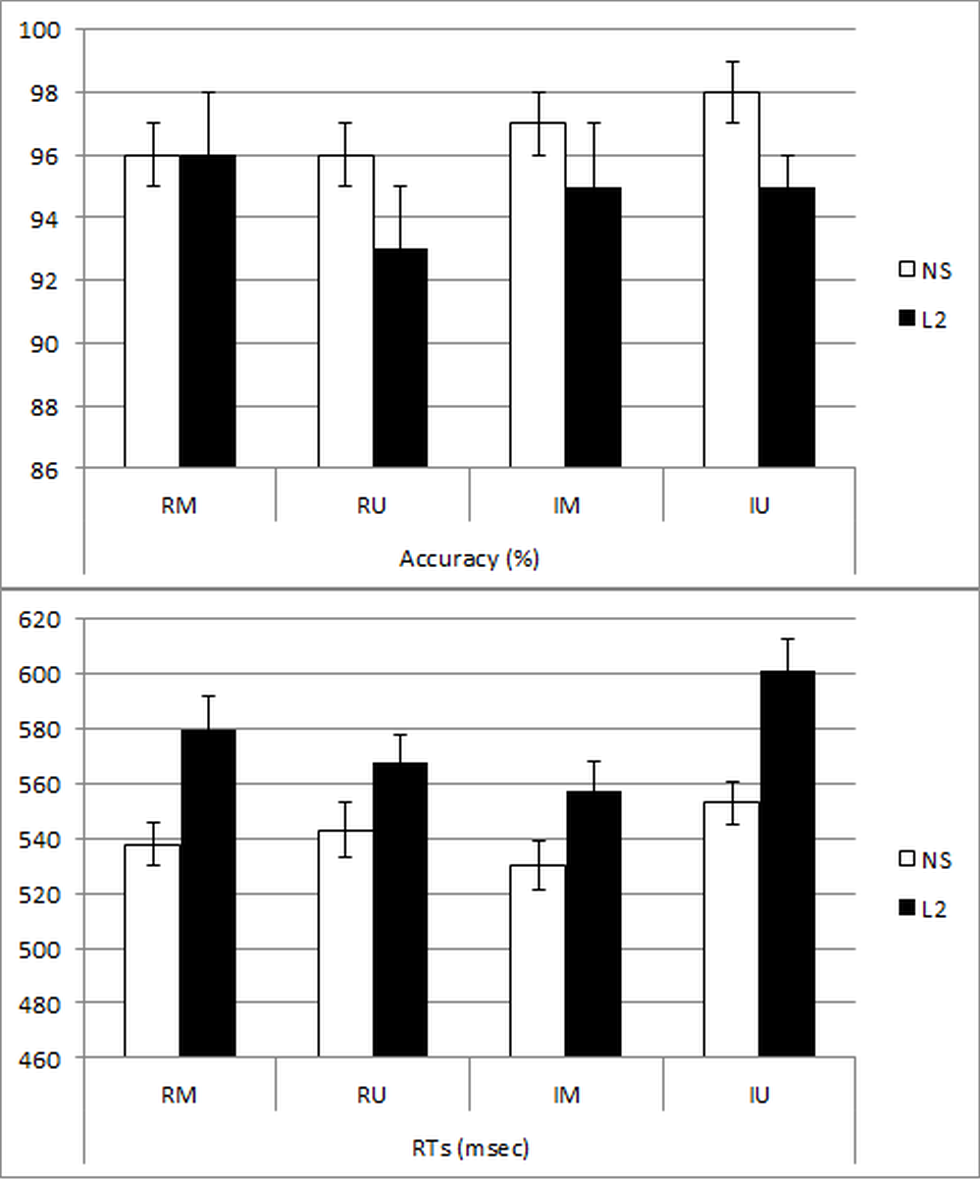

Supplement: Figure S1 — Behavioural results per group and per condition. Top: Accuracy (%), bottom: Reading times (msec). The error bars represent the standard error of the mean. (TIF) [file pone.0097298.s001.tif]
