# Supplementary material for: fMRI Evidence for the Involvement of the Procedural Memory System in Morphological Processing of a Second Language
Source: PLoS One. 2014 May 12;9(5):e97298. doi: 10.1371/journal.pone.0097298 (PMC4018348; doi:10.1371/journal.pone.0097298)
Supplement: Text S1 — fMRI whole-brain analysis. (DOCX) [file pone.0097298.s002.docx]

Our whole-brain analysis was performed in an identical way to that of the ROI analysis, with the exception of the masks. Similarly to our ROI analysis, we found no significant between-groups effects, therefore, we collapsed the two groups together into one single group. Table S1 illustrates the significant activations per condition for the combined group.

Similarly to our ROI analysis for the RM>IM contrast, we found significant activations in the LIFG and bilateral caudate nucleus. The right cerebellum did not emerge in this analysis; however, we found activation of the left cerebellum for the same contrast, suggesting bilateral involvement of the cerebellum in morphological processing. Interestingly, the whole brain analysis also gave us significant activations in areas such as the RIFG, bilateral occipital cortex, the left temporal lobe and the right paracingulate gyrus. These findings are not unusual in studies of morphological processing; for example, Tyler and colleagues (2005) reported activation of the bilateral temporal gyrus, the anterior cingulate, as well as parietal regions for auditory processing of regular vs irregular past tense forms. They suggested that processing of regular inflection engages an extensive fronto-temporal network, compared to processing of irregular inflection. This interpretation is not incompatible with our results, especially since we also found activations outside the procedural network and within these areas. Similarly, Bozic and colleagues (2010) reported significant activations in the RIFG for regularly inflected past tense verbs vs. simple forms that did not contain potential embedded stems (for example the form *trade* contains the potential stem *tray*). This effect is also applicable to our data, since irregularly inflected forms such as *kept* or *ate* do not include potential embedded stems. Still, this effect may not be related to processing of the *morphology* but the *phonology* of the presented forms, and as such, it is not directly related to the DP model. The large effects in the occipital cortex are harder to explain; however, since this region is concerned with visual processing, the differences for RM>IM maybe related to the length differences between the primes of each condition (the regular primes were longer than the irregular ones because of the *–ed* suffix). These effects are also repeated in the RM>RU contrast, where the significant difference in the prime length also applied. This contrast also produced bilateral activation of the IFG, with the LIFG activated in a region adjacent to the one that emerged in the ROI analysis. Finally, IM seems to have activated the right lingual gyrus more than both RM and RU. This is also an unusual effect, especially since this area does not normally emerge in this kind of experiment. We believe this may be due to the form properties of the irregular primes; however, this speculation needs further investigation.

Bozic, M., Tyler, L. K., Ives, D. T., Randall, B., & Marslen-Wilson, W. D. (2010). Bihemispheric foundations for human speech comprehension. *Proceedings of the National Academy of Sciences of the United States of America*, *107*(40), 17439–44. doi:10.1073/pnas.1000531107

Tyler, L. K., Stamatakis, E. A., Post, B., Randall, B., & Marslen-Wilson, W. D. (2005). Temporal and frontal systems in speech comprehension: an fMRI study of past tense processing. *Neuropsychologia*, *43*(13), 1963–74. doi:10.1016/j.neuropsychologia.2005.03.008

Table S1: Significant activations for the combined group in the whole brain analysis.

| **Contrast** | **hemi** | **region** | **Cluster sizea** | **Z** | **x** | **y** | **z** |
| --- | --- | --- | --- | --- | --- | --- | --- |
| RM>IM | R | Inferior lateral occipital cortex | 1688 | 3.75 | 46 | -70 | -8 |
|  | R | Precentral gyrus | 1470 | 3.86 | 48 | 2 | 46 |
|  | R | caudate | 1460 | 4.06 | 16 | 0 | 16 |
|  | R | Supplementary motor cortex | 1451 | 3.74 | 8 | 2 | 50 |
|  | R | Superior parietal lobule | 1347 | 4.06 | 34 | -52 | 58 |
|  | L | Inferior lateral occipital cortex | 897 | 4.47 | -46 | -70 | 2 |
|  | L | cerebellum, Crus II | 653 | 3.66 | -38 | -72 | -44 |
|  | L | Temporal pole | 470 | 4.08 | -50 | 8 | -4 |
|  | L | caudate | 461 | 3.21 | -22 | 8 | 24 |
|  | L | IFG operc | 332 | 3.4 | -40 | 6 | 26 |
|  | R | Paracingulate gyrus | 265 | 3.23 | 14 | 20 | 32 |
| IM>RM | R | Lingual gyrus | 1505 | 4.39 | 12 | -80 | -10 |
| RM>RU | R | Inferior lateral occipital cortex | 1195 | 4 | 40 | -78 | 6 |
|  | R | IFG operc | 873 | 3.73 | 46 | 6 | 22 |
|  | L | IFG triang | 631 | 3.34 | -40 | 24 | 16 |
|  | L | Inferior lateral occipital cortex | 476 | 3.37 | -40 | -70 | 2 |
|  | R | Thalamus | 391 | 3.77 | 8 | -8 | 8 |
| IM>IU | R | Lingual gyrus | 3498 | 4.83 | 14 | -84 | -10 |

All coordinates in MNI space

^a^Cluster size is expressed in number of 3x3x4 mm voxels

^b^IFG oper: Inferior Frontal Gyrus, pars opercularis
